# Microbial Siderophores: A New Insight on Healthcare Applications

**DOI:** 10.34133/bmef.0112

**Published:** 2025-03-21

**Authors:** Shilpa Borehalli Mayegowda, Manjula Nagalapur Gadilingappa

**Affiliations:** ^1^ School of Psychological Sciences, CHRIST University, Kengeri Campus, Bengaluru, Karnataka, India.; ^2^ School of Basic and Applied Sciences, Dayananda Sagar University, Bengaluru, Karnataka, India.

## Abstract

Globally, increased illness and disorders have gained importance in improvising therapeutics to help extend the lifespan of an individual. In this scenario, understanding the mechanism of bacterial pathogenicity linked to the interaction between the host and the pathogen focusing on essential metal ions is necessary. Numerous studies indicate that the severity of a disease might be due to the reduced availability of iron, linked to abnormal production or lack of acquisition systems. However, several microbes produce siderophores as virulence factors, low-molecular-weight organic compounds for acquisition of iron by iron-chelating systems. In medical applications, siderophores are employed in novel strategies in order to design effective new drugs and vaccines, targeting and delivering antibiotics to target sites in multidrug-resistant pathogens. Meanwhile, some types of siderophores are used as drug delivery modalities and antimalarial, anticancer, and antibacterial agents, for example, by employing conjugation techniques such as Trojan horse delivery. Hence, the current review integrates several applications of siderophores with an overview covering taxonomy, organisms producing iron affinity carriers, and their acquisition mechanism. This understanding may delineate newer opportunities to adapt possible therapies and/or treatments against several multidrug-resistant pathogens, representing a crucial solution for public health problems worldwide.

## Introduction

The fourth most abundant transitional element, iron, is found in the earth’s crust in oxidation states, as Fe(II) and Fe(III), with available forms as nonacidic, aqueous, oxygenated, iron oxides, and hydroxides. It is a crucial element in the chemical reactions of all cells for specific biological activities and the functioning of diverse cellular processes such as DNA metabolism, protein functioning, and synthesis of fatty acids. However, higher concentrations in humans can be lethal as oxidative stress leads to myocardial infarctions, pancreatic inflammation of adipose tissues, and liver damage [1]. Microbes have the potential to chelate iron by siderophores from the host cells for the uptake of iron. These siderophores are low-molecular-weight organic compounds produced by different microbial strains that help them to procure iron from the environment under deficient conditions. The physiological parameters of the environment such as pH present insoluble iron in the form of ferric ions (Fe^3+^), making it inaccessible, triggering the production of siderophores. However, they have gained immense importance in varied fields, like in the food industry, in agriculture for plant growth, in inhibition of phytopathogens, and as heavy metal detoxifiers; in the medical field, they have been used for delivering antibiotics to the targeted area in the “Trojan horse strategy”.

As an essential nutrient, iron is scavenged by both pathogens and commensals when they colonize the host cells. Microbes assimilate iron such as heme, ferritin, and transferrin through several pathways from the host. The most common is the scavenging pathway for the uptake of extracellular sources across the bacterial cell membrane with siderophore biosynthesis along with the regulation and expression of efflux and transport proteins [2]. A wide range of iron-related diseases have been efficiently treated by iron-chelating siderophores; their complexation with drug delivery systems has paved the way for the treatment of hydrocephalus, Parkinson’s disease, hepatitis, and delayed wound healing. Siderophores have also been used for inhibiting tumor growth by disrupting iron-related pathways in neoplastic cells. Subsequently, binding or chelating with other metals has allowed their use in molecular imaging by further incorporating with radionuclides, actinide decontamination, etc. [3].

## Classification of Siderophores

**Fig. 1.**
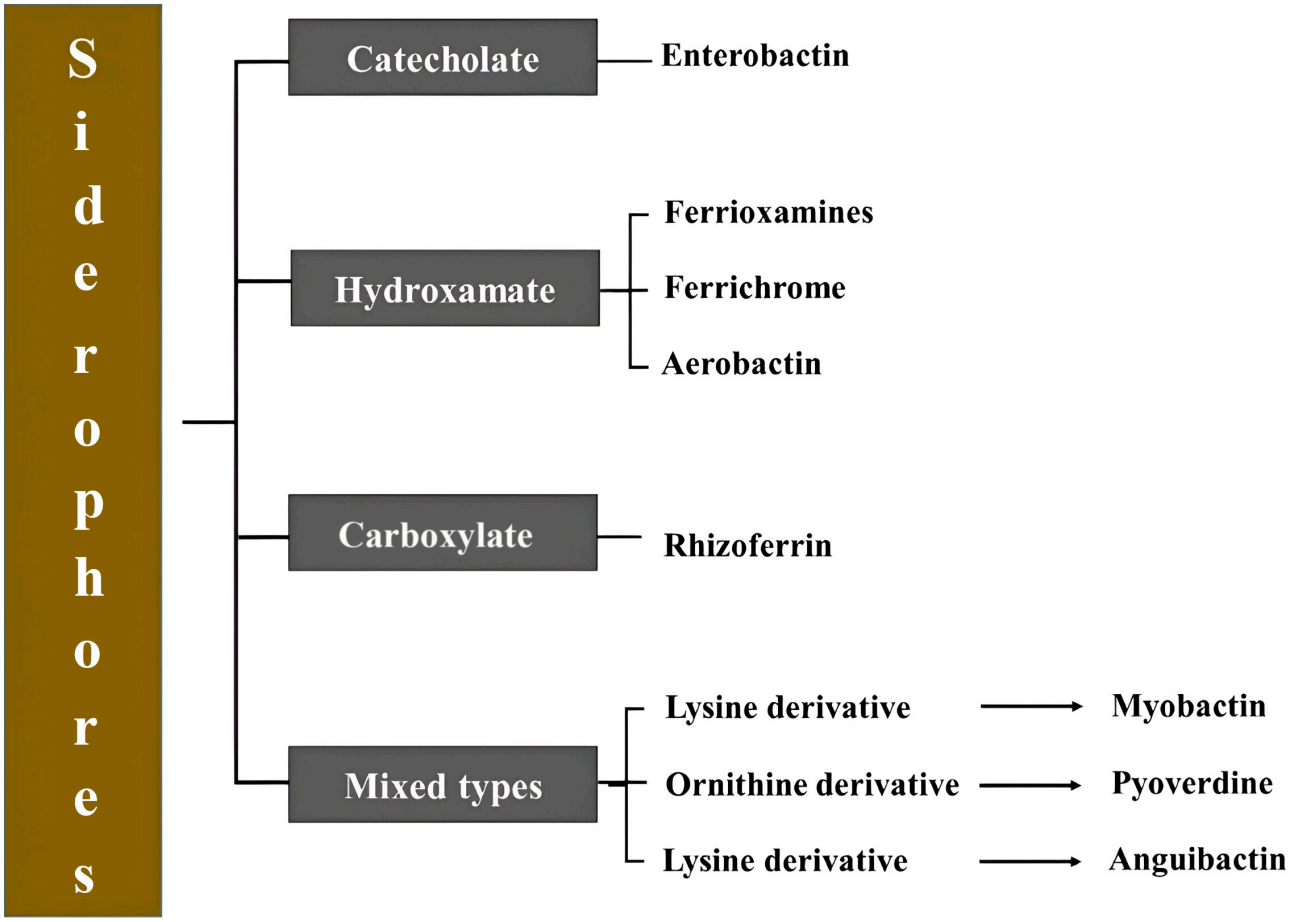
Classification of siderophores: catecholate, hydroxamate, carboxylate, and mixed types derived from different microbial species concerning their structure.

Over 500 types of microbial siderophores have been extensively studied and documented, with substantial variation concerning structural differences from species to species. The classification has 3 primary forms: hydroxamates, catecholates, and carboxylates (Fig. 1). Catecholates include enterobactin by *Streptomyces*, vibriobactin by *Vibrio cholerae*, and pyochelin by *Pseudomonas aeruginosa*, while hydroxamates are derived from *Alcaligenes denitrificans* (alcaligin), *Staphylococcus* spp. (staphyloferrin), *Mycobacterium tuberculosis* (mycobactin), and *Bacillus anthracis* (petrobactin). Generally, these siderophores are secreted in the environment and are water-soluble, and their presence in the media can be detected, as the medium color changes when they are produced. A definitive example is pseudomonads, which secrete yellowish-green pyoverdine and pyochelin, which are unique fluorescent siderophores produced during the scarcity of iron [4]. Similarly, fungi produce hydroxamate from the ferrichrome family, with few synthesizing carboxylates and phenolate [5,6]. However, *Saccharomyces cerevisiae* does not form siderophores but has a unique way of capturing iron from surrounding microbes using a siderophore–iron uptake system.

## Identification, Extraction, and Characterization of Siderophores

The most common detection methods for siderophore production include the Chrome Azurol S (CAS) assay, based on the competition for iron between the ferric complex of the dye indicator and the siderophore/chelator formed by the microbes. The positive reaction is the change from blue to orange color of the CAS reagent, due to iron removal from the media by the siderophores due to their high affinity for iron(III). The production of siderophores from the environment is quite challenging, as they are highly complex as they are present in soil matrices. However, extraction and purification can be a reliable resource as given by Egbers and co-workers. Numerous techniques have been used, such as ion-exchange chromatography, reversed-phase chromatography, size-exclusion chromatography, and solid-phase extraction [7]. Because these approaches had low recovery rates, liquid chromatographic techniques along with mass spectroscopy were employed to help purify and identify siderophores [8]. These techniques with limitations due to improper results of the complex mass and reduced intensities paved the way for immobilized metal affinity chromatography (IMAC) for purification of siderophores based on metal-ion affinity. Fe(III)-immobilized IMAC columns are used for the extraction and purification of siderophores. Metal oxide-based affinity chromatography has been used as a potential way of extracting metal ligands. The purified siderophores can be characterized using Fourier transform infrared analysis to determine the functional groups [9]. Other methods include nuclear magnetic resonance (NMR) spectroscopy to help identify the structure similar to synechobactins, orchobactins, and acinetobactin as observed in the spectral analysis of chryseochelin A. The structure is markedly aligned with heteronuclear multiple-bond correlation and nuclear Overhauser effect spectroscopy [10]. X-ray diffraction for metal complexation analysis and high-performance liquid chromatography (HPLC) with ultraviolet detection help characterize siderophores. Reversed-phase HPLC–mass spectrometry (MS) has observed the shortest retention time for hydrophilic siderophores. Additionally, liquid chromatography (LC)–tandem MS helps in the identification of siderophores with fragments represented by peaks. Although siderophores have been characterized by mass spectroscopy using LC–MS and gas chromatography–MS, there are other techniques used, such as electrospray ionization (ESI)–MS, matrix-assisted laser desorption/ionization (MALDI)–MS, inductively coupled plasma MS, and MS imaging [11].

## Microbial Mechanism by Siderophores for Iron Uptake

Iron is not accessible directly from the environment; rather, it is oxidized under aerobic conditions as an insoluble oxyhydroxide polymer with subsequent reduction to functional usage. Microbes have considerably diverse forms of iron transporters, which are species specific and uniquely adapted to synthesize and acquire iron by iron-chelating molecules, siderophores, which are low-molecular-weight compounds (<10 kDa), under specific conditions. Several microbial siderophores are acclimatized for adequate transport of nonavailable forms [6].

### Bacterial siderophores

In Gram-negative bacteria, the TonB-dependent outer membrane receptor (OMR), an adenosine triphosphate (ATP)-binding cassette (ABC)-type ATP-dependent, periplasmic siderophore-binding protein (SBP), plays a necessary role, while in Gram-positive bacteria, the lack of outer membrane makes it possible for SBP to be anchored to the extracellular membrane-associated ABC permease to recognize and import Fe^2+^ ions. The high-affinity siderophores ensure the continuous export of extracellular ferric iron recognized by OMRs and SBPs to form a chiral ferric–siderophore complex [12]. Plants also require iron for nutrition and development. However, lower concentrations affect photosynthesis and chlorophyll biosynthesis, hindering plant growth. Several microbes associated with plants like *Bacillus*, *Pseudomonas*, *Azotobacter*, *Enterobacter*, *Serratia*, *Azospirillum*, and *Rhizobium* have evolved mechanisms to respond to environmental uptake of iron molecules by siderophores for their metabolism, growth, and survival capabilities. Some microbes produce more than one siderophore, favoring other microbes for iron and other metal acquisition. These microbial siderophores have gained application as an alternative remedy in agriculture, medicine, and food, such as alleviating iron deficiency, serving as bioremediating agents, and other environmental aspects (Table 1). Their extensive application includes remediating metal contaminant levels in food and water, biocontrol of fungal pathogens, increasing soil fertility, and Trojan horse strategy for selective drug delivery to combat multidrug-resistant (MDR) pathogens [13,14].

**Table 1.** Various classes of siderophores by microbes and their applications in the medical field

Type	Siderophores	Microorganisms	Application in healthcare	References
Mixed type with lysine derivative	Aerobactin	*Escherichia coli*	Inhibition of the opportunistic pathogen *Klebsiella pneumoniae*, used for treatment against drug-resistant strains	[73]
Mixed type (serine protease inhibitor)	Aeruginosin	Cyanobacteria	Protein inhibitor, acts by inhibiting serine protease, can be used to block thrombosis	[74]
Catecholate	Arthrobactin	*Arthrobacter* spp.	Used as an iron chelator, to treat or overcome iron toxicity	[24]
Hydroxamate	Coprogen	*Pseudomonas chrysogenum*, *Histoplasma capsulatum*, *Neurospora crassa*, *Penicillium nalgiovense*	Produced by fungi and bacteria as an iron chelator, which inhibits the growth of *Candida* spp. and bacterial pathogens	[6,24,75]
Hydroxamate	Desferrioxamine B	*Streptomyces pilosus*	Used to minimize iron-induced toxicity by removing excess iron content in the blood in transfusion-dependent hemoglobin disorders	[76]
Hydroxamate	Dimerium acid	*P. chrysogenum*, *H. capsulatum*	Used to detect antisiderophores by conjugating with bovine serum albumin, helping in the identification of other siderophores	[77]
Hydroxamate	Ferrichrome	*P. chrysogenum*	Acts as antioxidant and heme-scavenging protein by reducing cytotoxicity in the vasculature by reducing low-density lipoprotein oxidation	[24,75]
Hydroxamate	Ferrioxamine E	*Streptomyces antibioticus*	Imaging and diagnosis by conjugating with ^68^Ga, a radioactive compound	[78]
Catecholate	Fusarinine	*P. chrysogenum*, *H. capsulatum*	Reduces oxidative stress and also acts as a heme-scavenging protein	[77]
Hydroxamate	Fusion	*Aureobasidium pullulans*	Antibacterial and anti-inflammatory	[79]
Hydroxamate	Mycobactin	*Mycobacterium tuberculosis*	Drug design and vaccine development by blocking mycobactin through its structural analog	[80]
Hydroxamate	Neocoprogen	*P. nalgiovense*	Bacteriostatic and antibacterial used in food processing and packing safety to maintain pathogen-free products	[24]
Hydroxamate	Ochrobactin	*Vibrio* spp.	Iron acquisition	[81]
Mixed type (ornithine)	Pyochelin	*Pseudomonas aeruginosa*	Imaging and therapy; conjugated with probes and antibiotics and can be used in biosensors and treating immunosuppressed patients, respectively	[25]
Mixed type	Sideromycin	*Streptomyces* spp.	Iron chelation and antimicrobial activity make it a potential therapeutic tool to develop a Trojan horse	[79]
Hydroxamate	Triacetylfusarinine	*Saccharomyces cerevisiae*, *P. nalgiovense*	Radiopharmaceutical imaging agents and diagnosis for identifying and treatment of fungal infection by multimodality imaging fluorescent dyes and antifungal moieties	[24,78]
Catecholate	Vanchrobactin	*Vibrio anguillarum*	Antimicrobial; an analog of vanchrobactin conjugated with antibiotic norfloxacin is used against *V. anguillarum* and its mutants	[82]
Catecholate	Vibriobactin	*Vibrio cholerae*	Development of vaccine and drug delivery can be done by conjugating with bovine serum albumin and immunized mice to produce antibodies and antibiotics, respectively, which can be used to treat cholera	[83]
Mixed type (nonribosomal peptide–polyketide)	Yersiniabactin	*Yersinia pestis*	Iron acquisition and drug delivery as a yersiniabactin–antibiotic conjugate help mediate cell entry through siderophore receptors and target with a spectrum of highly selective antibiotics	[83]
Carboxylate	Corynebactin	*Corynebacterium diphtheriae*	Iron acquisition and antibacterial as structurally related to staphyloferrin A from *Staphylococcus aureus*; the d-ornithine with a central lysine residue is linked through its α- and ε-amino acids by amide bonds to the carboxyl groups of 2 different citrate residues, thereby combating pathogens	[84]
Carboxylate	Staphyloferrin A Staphyloferrin B	*S. aureus*	Bacterial imaging studies and biosensors, as staphyloferrin A conjugates have structural factors and the stereochemistry of the amino acid backbone specifically and efficiently target *S. aureus*; fluorescent staphyloferrin A probes specifically target staphylococci in complex bacterial mixtures	[85]

**Fig. 2.**
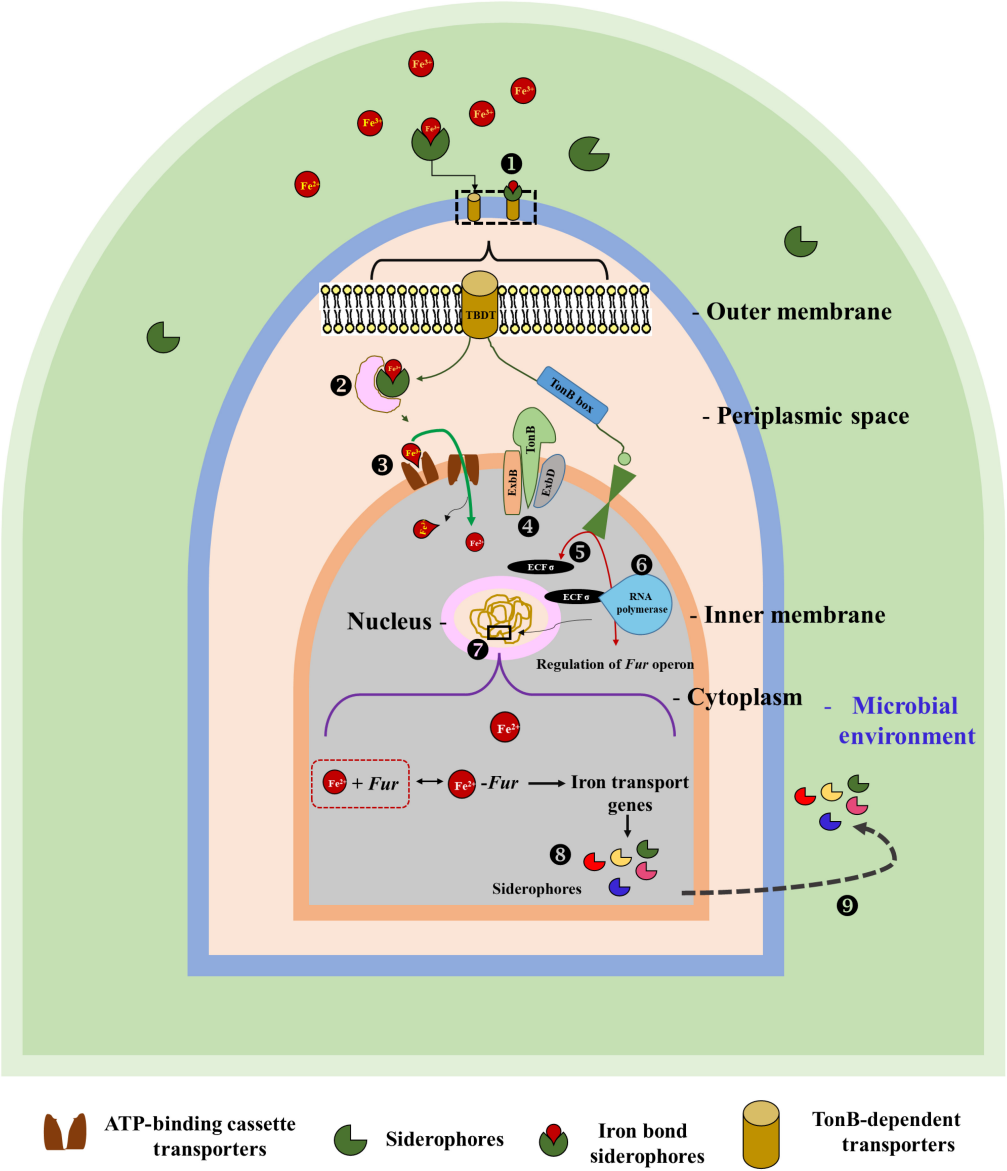
Transportation of iron across the outer and inner membranes of the microbial cell. (1) The outer membrane has receptor-binding proteins (TonB). (2) The siderophore–iron complex (SIC) in periplasmic space. (3) The SIC binds periplasmic binding proteins and gets transported with the help of adenosine triphosphate (ATP)-binding cassette (ABC) transporters. (4) The ExbB and ExbD complexes help the SIC across the outer membrane. (5) ExbB–ExbD–TonB activates extracellular fluid protein sigma (ECFσ) for activation of RNA polymerase. (6) RNA polymerase activates the *Fur* (ferric uptake regulation) operon. (7) The *Fur* box helps transcribe *Fur* proteins. (8) Specific types of siderophores are formed by specific microorganisms. (9) Siderophores are released outside the cell for the uptake of iron from the surroundings inside the cells. TBDT, TonB-dependent transporter.

Many microbes are extremely sensitive to fluctuating levels of iron. In contrast, siderophore-mediated iron is an indicator by other organisms in their vicinity to modify their physiology and development as the producer organism. The most prevalent mechanism for iron acquisition is through the *Fur* (ferric uptake regulation) box regulator, which regulates ferric uptake by an iron-concentration-dependent protein (Fig. 2). Siderophore production is inhibited at higher iron concentrations as the *Fur* protein binds to the promoter gene; i.e., the *Fur* operon remains off. It becomes functional only during reduced intracellular iron concentration with modified conformational changes in the *Fur* protein, causing it to detach from the promoter and inducing siderophore production. Eventually, a free siderophore from the promoter region is released out of the host cell; binds to the iron moiety of Fe(II) or Fe(III) form, mediated with the help of membrane receptors, periplasm membrane proteins, and TonB receptors; and is transported back inside the cells. Upon entering the host cells, the iron moiety is reduced from ferric to ferrous ions [15].

Additionally, microbes have iron-acquisition systems along with siderophores, such as hemophores and heme systems [16]. However, remarkable differences lie between Gram-negative and Gram-positive bacteria as regards the siderophore regulatory mechanism for mediating Fe transport. In Gram-negative *Escherichia coli*, this mechanism is mediated by TonB-dependent OMRs that recognize Fe(III) and bind to siderophores, forming an Fe(III)–siderophore complex. Further, the complex binds to periplasmic binding proteins and gets released into the periplasmic space. With the help of an ABC transporter, it gets transported across the cytoplasmic membrane to reach the cytoplasm, with the iron in the siderophore complex reduced from Fe(III) to Fe(II). In constrast, *Bacillus* spp. lack an outer membrane, hence the absence of OMRs. Therefore, Fe(III)–siderophore complexes bind to cell membrane receptors through periplasmic siderophore protein binders as they lack periplasmic space and are transported to the cytoplasm by ATP-dependent transporters [17].

However, in Gram-negative bacteria, outer membrane TonB receptor-binding proteins form a siderophore–iron complex, which is transported through the periplasmic space to the cytoplasm by a protein channel with the help of ABC transporters. Further, ExbB and ExbD, inner membrane protein complexes, help transport the siderophore–iron complex across the outer membrane. ExbB–ExbD–TonB activates extracellular fluid protein sigma, responsible for activating RNA polymerase, which remains accountable for activating the *Fur* operon to regulate iron uptake. As regards the regulation of genes, the *Fur* box helps transcribe *Fur* proteins at low iron concentrations and increases the uptake while preventing high concentrations by forming a *Fur*-and-Fe^2+^ complex (Fig. 2).

### Fungal siderophores

The fungal siderophore has 4 diverse and unique mechanisms for Fe transport systems. The first includes the shuttle mechanism observed in *Ustilago maydis*, where the Fe(III)–siderophore intricate complex gets transported across the cell membrane, releasing Fe(III) into the cytoplasm by reductive enzymes, with siderophores being recycled back [6]. Secondly, in the taxicab system in *Rhodotorula* spp., the Fe(III) from the extracellular siderophore is transported to the intracellular ligands across the cell membrane [5]. The third comprises a hydrolytic mechanism involving the transport of the whole Fe(III)–siderophore intricate complex inside the cell by undergoing numerous degradative and reduction reactions forming Fe(II) and expulsion of siderophores as observed in Mycelia Sterilia for the uptake of the Fe(III)–triacetylfusarinine complex [18]. The last comprises a reductive pathway, with little deviation from other types in which transporters are absent for the Fe(III)–siderophore complex with the reduction of Fe(III) to Fe(II) occurring at the cell membrane. For example, *Ustilago sphaerogena*, a smut fungus, reveals Fe(III) uptake from ferrichrome and cotransport of zinc and cobalt. Although iron deficiency is a crucial factor in the biosynthesis of siderophores, external parameters such as temperature, pH, metals, carbon, and other nutritional limits play an essential role [5]. A study by Valdebenito and team [19] revealed that an *E. coli* strain had shown heightened production of hydroxamate such as aerobactin at low acidic pH levels. This is in contrast to microbes forming catecholate like yersiniabactin and salmochelin at neutral to alkaline pH levels.

## Application of Siderophores

### Probiotics

The applications of siderophores in varied fields have been researched, such as food production and processing, to enhance the nutritional quality of food products. They regulate enzymes with iron as a cofactor for various physiological functions like respiration, oxygen transport, and DNA and amino acid synthesis. The iron used in food fortification may be insoluble, but with the help of siderophores, producing colon-targeting probiotic microbes would have the best effect on human health. They possibly help correct the iron deficiencies that might lead to anemia and cancer by restoring the iron required for metabolic processes like the formation of red blood cells and repair of DNA. The rapid multiplication of these probiotic microbes is due to their unique ability to overcome iron deficiencies [20]. A study highlighted the production and characterization of siderophores from probiotics to treat anemia from a traditional fermented food called chhurpi (a Himalayan fermented food), wherein it was found that *Bacillus subtilis* L9 produces catecholate and *Pediococcus pentosaceus* BAC L7 synthesizes hydroxamate. Their purified siderophores exhibited a high iron-scavenging activity, highlighting the presence of probiotic microbes in indigenous fermented food with effective therapeutic options for extreme iron absorption in mitigating anemia and also combating high-altitude hypoxia [21].

A study on the production of deferriferrichrysin siderophores from *Aspergillus oryzae* evidenced the role of antioxidants in balancing reduction/oxidation in food products. In addition, a decade back, Todokoro et al. [22] proved the beneficial role of *Bacillus* spp. as a probiotic with the production of 2,3-dihydroxybenzoic belonging to the class of catecholate with antimicrobial properties [22]. The survival of *Bacillus* in extreme conditions and its adaptation has been employed in the gastrointestinal tract to eliminate some enteric pathogens, like *Salmonella typhimurium*, *Streptococcus pyogenes*, and *Staphylococcus aureus* [23]. Antimicrobial activity has been a contributing factor of probiotic bacteria against several pathogens; however, no mutants were generated to assess siderophore metabolism and functional responsibility as indicated by De Serrano [24]. Hence, it becomes imperative for researchers to establish the link between siderophores and novel probiotic bacteria to help create newer products in food industries.

### Biosensors in the pharmaceutical industry

**Fig. 3.**
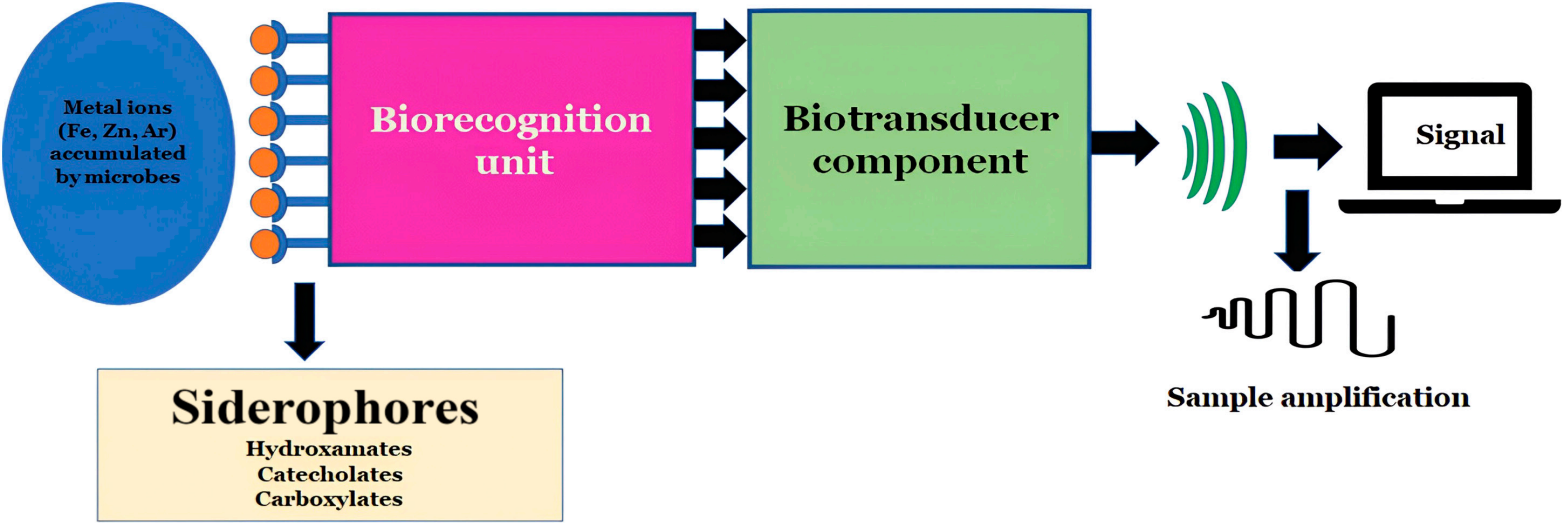
Schematic representation of biosensors with siderophores showing forms of iron–siderophore coordination complexes amplified and transduced to receive signals.

Like in other fields such as forensic science, pharmaceutical sectors, and molecular biology, the role of siderophore-based biosensors has gained immense popularity in food industries. A fluorescent pyoverdine siderophore derived from *P. aeruginosa* has been applied in biosensors to monitor and detect the iron elements from samples [25]. Pyoverdine-based biosensors are highly selective for Fe(III) detection and have been used to analyze water samples in trace amounts either in solution (10 ng/ml) or in immobilized form (3 ng/ml) (Fig. 3). A study reports that due to their high effectivity and stability, these siderophore biosensors were used for over 3 months to conduct around 1,000 tests or more [26].

A derivative desferrioxamine (DFO) B, *N*-methylanthranyl DFO is widely used as an environmental chemosensor and a photoactive biosensor in aquatic systems [27]. The isolation of extremophilic aquatic microbe *Paracoccus denitrificans* has helped analyze iron concentrations from oceans as determined by the parabactin derived from it and helped transform and bioremediate metal bioleaching. The siderophores derived from *Pseudomonas fluorescens* have been well characterized with high sensitivity and specificity for user-friendly biosensors for detecting iron (Fig. 3). However, only a few potential biosensors have been used in applications, as research is needed to characterize siderophores for biosensors [28].

### Trojan horse antibiotics for combating bacterial infection

The rapid emergence, dissemination, and spread of MDR strains have threatened lives across the globe, which has led researchers to develop newer therapies and treatment options. The underlying situation can be achieved through a basic understanding via infection surveys and the preventive methods adopted worldwide to help manage healthcare-associated infections. Several investigators have demonstrated that MDR strains’ pathogenicity competes with iron bioavailability by chelating agents, which contribute to the development of infections during host–pathogen interaction and thus hinder treatment options. The major event during host–pathogen interaction includes stealing iron from the host and triggering host susceptibility, as these pathogens produce different kinds of siderophores, which helps them initiate a faster growth rate along with virulence factors resulting in pathogenicity and infection. These pathogenic siderophores belong to a family of ferroproteins like transferrin and ferritin, leading to the host’s weakness.

**Fig. 4.**
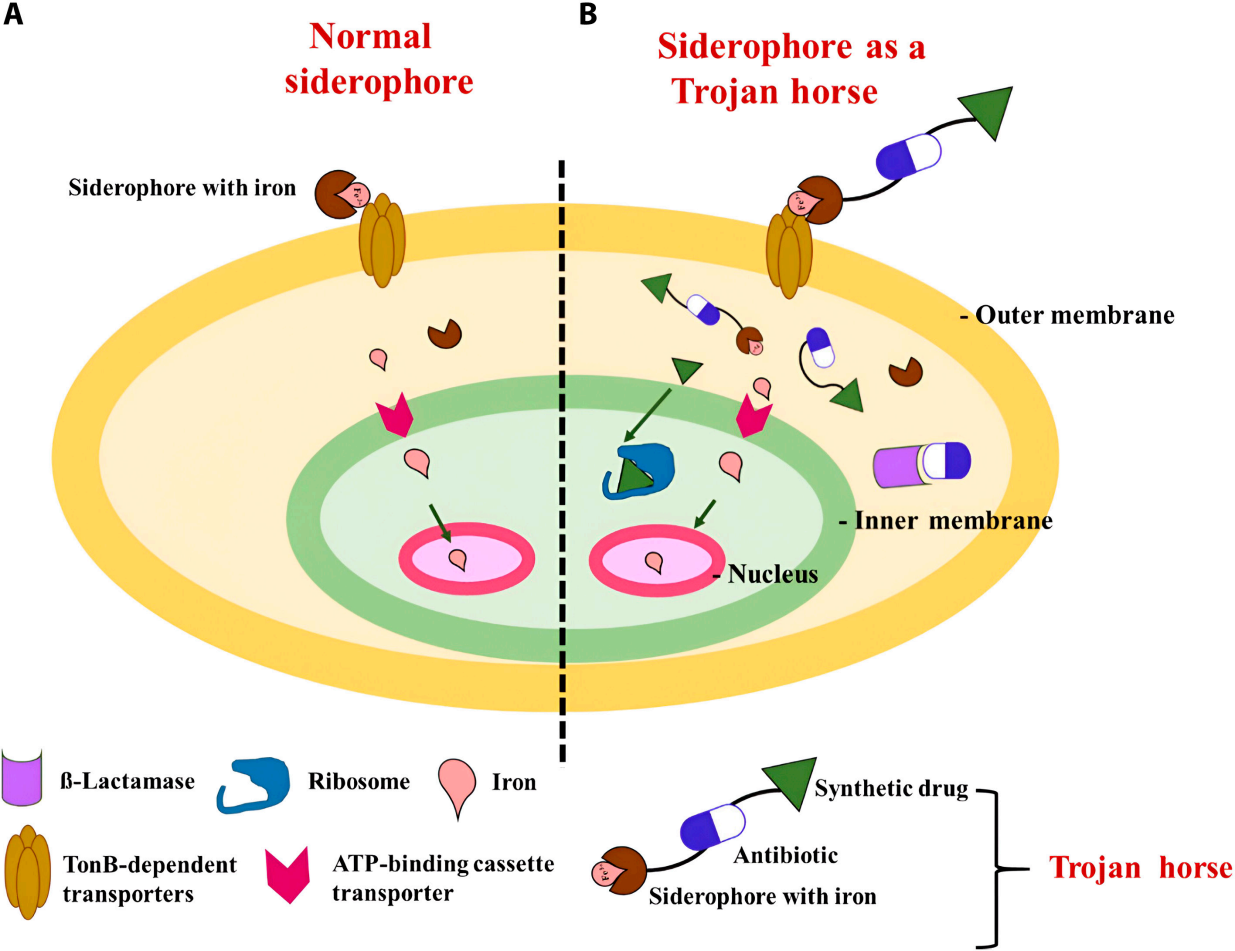
Application of siderophores as a Trojan horse. (A) In general, siderophores are activated at low iron levels and chelate iron from the surrounding environment and transport it to the cytoplasm for cell activities and metabolism. (B) The “Trojan horse strategy” process inhibits multidrug-resistant (MDR) strains by siderophore–antibiotic complexes through the spacer entering the cell using the siderophore receptors on the membrane. On entering a bacterial cell, the spacer gets hydrolyzed, releasing the antibiotics to target and cause cell death. The “Trojan horse strategy” is more effective with conjugated synthetic drugs along siderophores and antibiotics (synthetic drug–siderophore with iron–antibiotic).

**Fig. 5.**
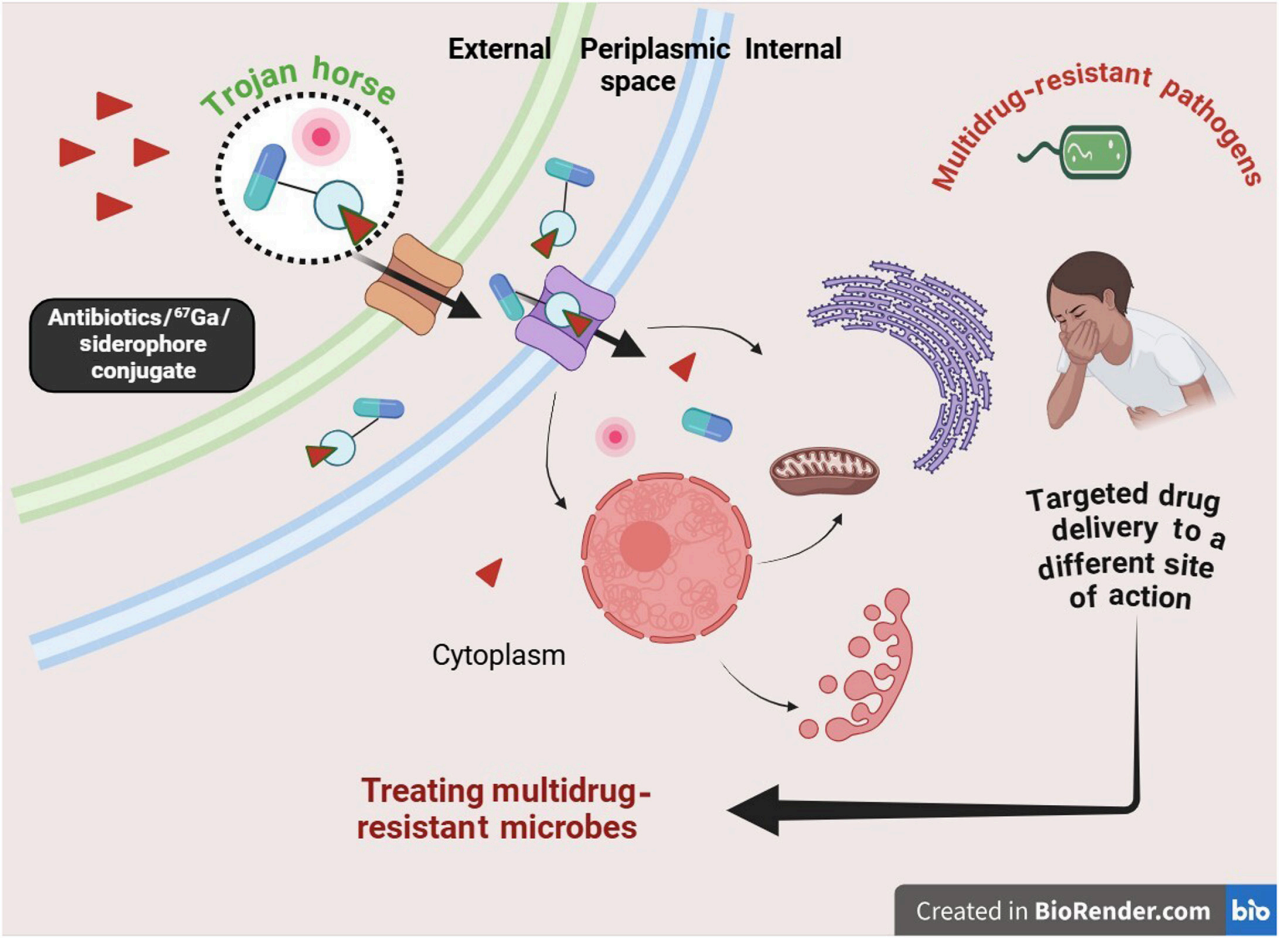
Antibiotic conjugated with siderophores delivers iron to the host to fight against pathogens. Also shown is the effect of antibiotics, which work by blocking the cell division of MDR pathogens through various ways.

The study of antibiotics with antibacterial activity against MDR pathogens has paved the way for an innovative novel bacterial iron-acquisition system to help enhance drug delivery inside the cells (Fig. 4). Gram-negative bacteria such as *E. coli* and *P. aeruginosa* produce siderophores such as enterobactin and pyoverdine that are similar to cefiderocol and can be substituted with the similar molecule catechol [29]. Cefiderocol, similar to siderophore, helps bind extracellular ferric iron that can be transported across the outer cell membrane by the iron transport system of the microbes. This mechanism helps cefiderocol overcome the carbapenem resistance mechanism in Gram-negative bacteria, such as efflux pump up-regulation and porin channel mutation. Cefiderocol, a beta-lactam antibiotic, binds to penicillin-binding proteins to inhibit cell wall synthesis in pathogenic bacteria (Fig. 5).

Antibiotics also confer stability through side-chain properties against the hydrolysis of β-lactamase enzymes of both extended-spectrum β-lactamases and metallo-β-lactamases. A study also showed the enhanced potency of antibiotics targeting RNA polymerase by using microbial iron transport machinery to help deliver and translocate across the cell membrane for MDR strains. The modification with a covalent bond resulted in moderate to low antibiotic activity. It was designed with cleavable linkers that helped release antibiotics in bacterial cells with tranquil targeted drug release (Fig. 4). The study utilized 10 cleavable siderophore–ciprofloxacin conjugates with chelators having systematic variations and a linker moiety to help identify quinone trimethyl to lock the conjugates as superior linker systems. Further, 3 structurally and mechanistically different products of RNA polymerase inhibitors using rifamycin, sorangicin A, and corallopyronin were conjugated with a quinone linker to hydroxamate and catecholate siderophores, which increased the antibiotic activity against MDR *E. coli* to 32-fold as compared to free rifamycin alone [30]. Microbes play a substantial role in activating siderophores by their metabolites and enzymes, like the use of citrate synthase regulated by the SbnG gene, which helps provide citrate for staphyloferrin B in several pathogens like *S. aureus*. However, the SbnG structure closely resembles class II aldolases to catalyze the aldol condensation to ketose, a crucial step in the amphibolic pathway of bacteria [31]. As staphyloferrin B contributes extremely greater to pathogenesis, inhibitors against it can block SbnG citrate synthesis without affecting the host’s tricarboxylic acid cycle. Likewise, baulamycin A and B derived from *Streptomyces tempisquensis* are highly potent inhibitors of the siderophore biosynthetic pathway that has been reported in several pathogens like *E. coli* and *S. aureus* [32].

A treatment option for MDR pathogens causing infectious diseases has been researched with a promising approach by the use of “Trojan horse therapy”. The underlying mechanism also involves targeting the siderophore biosynthetic pathway to deny pathogens from accessing iron molecules. The “Trojan horse strategy” exploits siderophores’ iron-transporting capabilities to carry out selective antibiotic delivery mediated by siderophores to the pathogens by conjugated antimicrobial–siderophore–iron complexes. This conjugate also led to the use of synthetic antimicrobial–antibiotic–siderophore–iron complexes on MDR strains (Fig. 4) [33]. Gram-positive and Gram-negative bacterial inhibition by albomycin, a naturally occurring antibiotic conjugated with sideromycins, and its toxicity are identical to those of ferrichrome with affinity to a serine spacer, thereby entering through the ferrichrome uptake system and being released enzymatically [34]. Comparatively, Gram-negative bacteria are less sensitive than Gram-positive bacteria to antimicrobials due to their outer cell membrane having an efficient permeable barrier. Following antibiotic-conjugated siderophores that are efficiently transferred across the bacterial membrane, eradicating these infectious diseases has been an exciting area as a treatment option in medical field advancements that have been explored. The “Trojan horse strategy” is an efficient delivery system for antibiotics to the cell via the siderophore-uptake system instead of porin proteins (Fig. 5) [35]. The susceptibility of an MDR strain of *P. aeruginosa* toward β-lactam antibiotics is due to its narrow porin channels compared to those of other pathogens, increasing its drug resistance due to minimal antibiotic uptake [36]. However, synthetic siderophores along with β-lactam antibiotics complex have shown promising results for antimicrobial activity, with the mechanism of binding penicillin proteins in the periplasm and inhibiting Gram-negative pathogens [37].

Investigators have documented well the efficacy of clinically used conjugated antibiotic–siderophore complexes like MC-1 (monocarbam), BAL30072 (monosulfactam) [38], S-649266 (cephalosporin), and GSK3342830 (cephalosporin) [39] against the MDR strains of *Acinetobacter baumannii* and *P. aeruginosa*, respectively. The process of vectorization of siderophore–aminopenicillin has helped extend its application in treating cystic fibrosis and systemic infections caused by *P. aeruginosa* in an animal model with ED_50_ values with low toxicity as compared to using only ofloxacin per se [40]. Several biofilm-forming microbes, such as *P. aeruginosa*, are influenced by heightened pathogenicity and infectivity rate affecting iron depletion or by involvement of twitching motility and a quorum sensing system. Siderophores are used to compete with these pathogens for iron depletion, which retards the formation of biofilm and increases twitching motility with the expression of quorum-sensing-related genes in *P. aeruginosa* [41].

The emerging MDR strains of methicillin-resistant *S. aureus* and their associated infections have higher morbidity and mortality rates, leading to treatment option challenges. The salts of gallium, gallium maltolate, are known to be potential therapeutic agents used to combat methicillin-resistant *S. aureus*. The process of competitive binding of gallium (Ga^3+^) instead of iron (Fe^3+^) facilitates the arrest of metabolic activities mediating redox reactions in pathogens with zero hindrance in the host’s biological activities [42]. A similar study noted that the use of Ganite (gallium nitrate) for treating hypercalcemia has effective antimicrobial activity against *A. baumannii* in both in vivo and in vitro analyses [43]. An increased antibacterial and antibiofilm response by *Streptomyces pilosus* secreting DFO–gallium–siderophore and gentamicin (Ga–DFO–gentamicin)-conjugated siderophores was evidenced by their efficacy by correlation in rabbit animal models where topical administration decreased the severity of corneal infection caused by *P. aeruginosa*. Carboxylate siderophores, such as staphyloferrin A and staphyloferrin B, derived from *S. aureus*, are produced during extreme critical iron-limiting conditions for survival. The process of acid stress helps trigger the production of staphyloferrin. A hydrophilic situation improves water solubility to favor iron-chelating properties and to regulate the rate of transportation into the cell as compared to catecholate and hydroxamate [44].

Emergent fields, like metabolomics, have been used more frequently to target specific small molecules with extreme difficulty in treating MDR strains, specifically *M. tuberculosis*, which affects 10 million people worldwide. The production of mycobactin and the carboxylated form of carboxymycobactin can ease the rapid progression of such disease. In recent decades, the siderophores produced by *M. tuberculosis* have also been used as a target site in many pharmacological methodologies (Fig. 5) [45]. A study executed in an animal by blocking the 2,3-dihydroxybenzoate-AMP ligase (MBTA), an enzyme that forms adenylate, involved mycobactin biosynthesis by the MBTA inhibitor salicyl-AMS (5′-*O*-(*N*-salicylsulfamoyl)adenosine) [46]. A study also noted that inhibitors like benzimidazole-2-thione 4 (inhibitor of salicylate synthase MBTI) have potential activity in clinical settings, and the researchers also noted the need for further investigations [47]. However, it has been shown that blocking the action of mycobactin and carboxymycobactin exporting iron is one of the main functions of siderophores [48]. Hence, it opens a new window to siderophore target-based drugs to help give future directions in new-generation drug design (Fig. 5).

### Antimalarial and antituberculosis activity

**Fig. 6.**
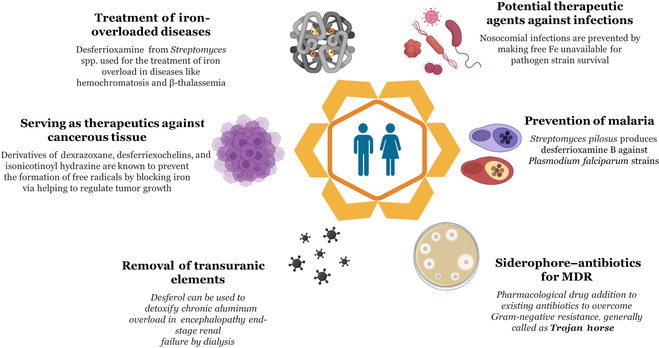
Several applications of siderophores in the medical field are used for targeting MDR pathogens to cure infections by using the “Trojan horse strategy”, treatment of iron-overloaded diseases, antimalarial treatment, anticancer therapy, and removal of transuranic elements from the system.

Siderophores have also extended their functional role in helping treat protozoan infections like malaria by possessing antimalarial activity against strains of *Plasmodium falciparum*. DFO B, secreted by *Klebsiella pneumoniae* and *S. pilosus*, showed antimalarial action against *P. falciparum* [33]. The mechanism of DFO B was depleting iron intracellularly by entering the parasitic cells. This siderophore–methyl anthranilic acid conjugate exhibited excellent activity more than 10 times against *P. falciparum* (Fig. 6). Increased activity was also observed against the same pathogen by forming a complex with a synthetic quinolone conjugate of nalidixic acid. Each conjugate of siderophore action draws a linear correlation in performing its function effectively by damaging the cell via DNA degradation by metal-catalyzed oxidative reactions. The conjugated siderophore is more effective as it vigorously weakens pathogens by producing many siderophores with antituberculosis activity against MDR *M. tuberculosis* strains. A study by Miller et al. [49] revealed that artemisinin, an herbal drug used to treat malaria, was effective as an antituberculosis agent when conjugated with a siderophore analog against *M. tuberculosis*-infected cells. Therefore, vectorization of the siderophore and its related components can be a promising therapeutic modality for treating such diseases, resulting in one appropriate potent conjugate with selective activity against 2 of the most fatal diseases in the world.

### Therapy for iron-loaded diseases

β-Thalassemia and sickle cell anemia patients require periodic/repeated blood transfusions for their treatment, resulting in the steady buildup of iron in their body system without any specific physiological mechanism to help remove it. However, overloaded iron must be minimized from the liver, which can be treated using siderophore-based drugs. Thalassemia major and sickle cell anemia are traditionally treated with Desferal to overcome iron accumulation in body fluids [50]. Gallium-loaded siderophores are recommended as gallium-based therapeutics, such as DFO (Desferal). Siderophores extracted from *S. pilosus* have been successfully used for treating iron-overload diseases like hemochromatosis and β-thalassemia (Fig. 6). They are vital as they can treat red-blood-cell-related diseases and/or abnormalities like malaria and sickle cell anemia [51].

### Therapy for transuranic element removal

The process of advanced diagnosis is incomplete or close to impossible without the use of nuclear energy. Nuclear radiation provides insight into the functioning of a person’s specific organs, helps clinicians with rapid diagnosis, and treats disease and abnormal cell growth. However, the increased use of transuranic elements like aluminum and vanadium adversely affects health. An increased aluminum accumulation in patients already suffering from end-stage renal failure and dialysis encephalopathy and repetitive exposure to medical devices working on nuclear energy generates an alarming concern in healthcare issues. However, no treatment options help remove this transuranic element from the body system, further deteriorating health conditions. In this scenario, siderophores are a boon that helps resolve the issue caused by transuranium elements. Desferal has a potential role in removing the overloaded iron and aluminum used to treat chronic conditions, following the mechanism of mobilization and chelation, binding aluminum ions with tissues, and forming an aluminoxamine complex. Further, this complex, being water-soluble, can be easily excreted in urine and feces. Desferal has also successfully removed another transuranic element, vanadium, in an animal model at rates of 20% and 25% from the kidneys and lungs, respectively [52].

### Therapy for cancers

Excessive iron deposition enhances iron-catalyzed free radicals, mediating oxidative stress and posing a high risk of cancers and increased mortality. It is well noted that cells require the iron–sulfur clusters found in hemoglobin or other proteins for normal cellular and physiological functioning. However, the problem arises when free-state iron becomes reactive, damaging the cells and leading to hemochromatosis and endometriosis, inducing cancers. Phlebotomy has been used as a treatment option in patients with peripheral arterial disease to reduce the iron content and the disease-causing severity. The prolonged existence of ovarian endometriosis (>10 years) may lead to ovarian cancer due to high levels of oxidative DNA damage of the epithelial cells catalyzed by overloaded iron forms of endometriotic cysts in the ovaries. The rapidly dividing cancer cells require a high concentration and uptake of iron as compared to healthier cells. The abnormal cells’ increased demand for iron must be blocked to abort the cell proliferation, which can be accomplished using iron chelators like siderophores, as they are beneficial for treating cancers [53]. DFO remarkably reduces aggressive tumors from proliferating further in patients with neuroblastoma or leukemia [54]. A history of DFO-mediated reduction in the cell viability of neuroblastic tumors of up to 80% was seen after 4 h of incubation. This study also showed remarkable inhibition of DNA replication from 4 to 72 h [55]. A study on the siderophore activity produced by *Actinobacterium*, DFO E, showed that it extensively helped the host reduce the viability of the malignant melanoma cells [56]. Other potent siderophore activities include aredexrazoxane, *o*-trensox, desferriexochelins, desferrithiocin, and tachpyridine in the treatment of cancer by acting as iron chelators. Currently, siderophores have been varyingly prolific in treating chemotherapy patients with increased serum levels of non-transferrin-bound iron. One of the potentials being sought is with deferasirox, which exhibits an effective antileukemic effect by ultimately reducing the suffering in a patient from chemotherapy-resistant acute monocytic leukemia [57].

### Siderophoregenic microbes used as medium enhancers

Usually, unknown microbes are identified by molecular data analysis of 16S ribosomal RNA genes, revealing >99% accuracy. This technique is expensive to use for environmental sample analysis, which involves huge numbers of samples, with the culture media used to count all kinds of samples not providing the actual distribution and abundance of the total population [58]. Therefore, it becomes incredibly challenging to culture samples to reveal some unknown microbes, as it is difficult to analyze the growth requirements on specific media. This situation can be overcome by using siderophoregenic microbes or siderophores that help favor growth when co-cultured or even cultured in the media enriched with siderophores. D’Onofrio et al. [59] stated the role of siderophores formed and produced by the bacterial cultures that played a vital role in the growth of the uncultured bacteria on the synthetic medium. Further, these uncultured bacteria can be recovered and modified on synthetic media with the help of uncultured bacteria and retrieved on siderophores, leading to the discovery of novel microbes. This culturing technique also helps analyze the results at a less cost than molecular analysis.

### Bacterial typing or siderotyping

As the production of a specific molecule, pigment, hormone, and enzyme is specific to any class of microbial species, likewise, specific siderophores are produced by siderophoregenic microbes. Many researchers use this specificity to identify unknown microbial strains, known as siderotyping. An example is the fluorescent pyoverdine produced by *Pseudomonas* spp., which is applied as a taxonomic marker to classify and identify the closely related strains in bacteria. This siderotyping also adapts techniques like MS and isoelectrophoresis for effective identification. However, additional research and development in this area are required to help develop a solid database to perform the technique efficiently [60].

A genomic and functional characterization of a gene cluster in the bacteria *Burkholderia xenovorans* containing a nonribosomal peptide synthetase revealed its role in the production and transportation of siderophores. With the lack of iron, the strain produced a siderophore identified by ESI–MS and MALDI/time-of-flight–MS studies as a type of peptide not produced by ribosomes and can bind with iron to form a complex with a molecular mass of 676 Da [61]. Accordingly, from bioinformatics prediction, CAS assay, and MS study, it was identified as L-*N*-d-hydroxy-*N*-d-formylornithine-D-b-hydroxy-Asp-LSer-L-*N*-d-hydroxy-*N*-d-formylornitine-1,4-diaminobutane. Genome mining was employed to assess different strains for the existence of genes associated with lipopeptide siderophore production as variochelin A and B, which are synthesized by the enzyme polyketide synthase, derived from terrestrial bacteria *Variovorax boronicumulans* and characterized using NMR and MS techniques, together with Wu et al.’s study [62]. The identification of a new group of catecholate hydroxamate siderophores called qinichelins from *Streptomyces* sp. was made possible by genome sequencing and advancements in bioinformatic technologies. The correlation between the amounts of metabolites and protein expression profiles has discovered the biosynthetic gene cluster qch, most likely responsible for manufacturing qinichelin [63]. The molecular structure was determined using bioinformatics, MS, and NMR. The synthesis of siderophores by any microbe requires the lack of or extremely low iron levels in the growth medium as in *Synechococcus* sp., the media included 50 nM FeCl_3_·6H_2_O. A low concentration of Fe^2+^ ranging from 15 to 100 mM is used to detect the siderophore fusigen [27] from the marine yeast-like fungus *Aureobasidium pullulans* using HPLC–MS/ESI with a yield of 1.1 mg/ml and exhibiting antibacterial properties against *Vibrio* bacteria from diseased marine species [64].

### Siderophores as vaccines

**Fig. 7.**
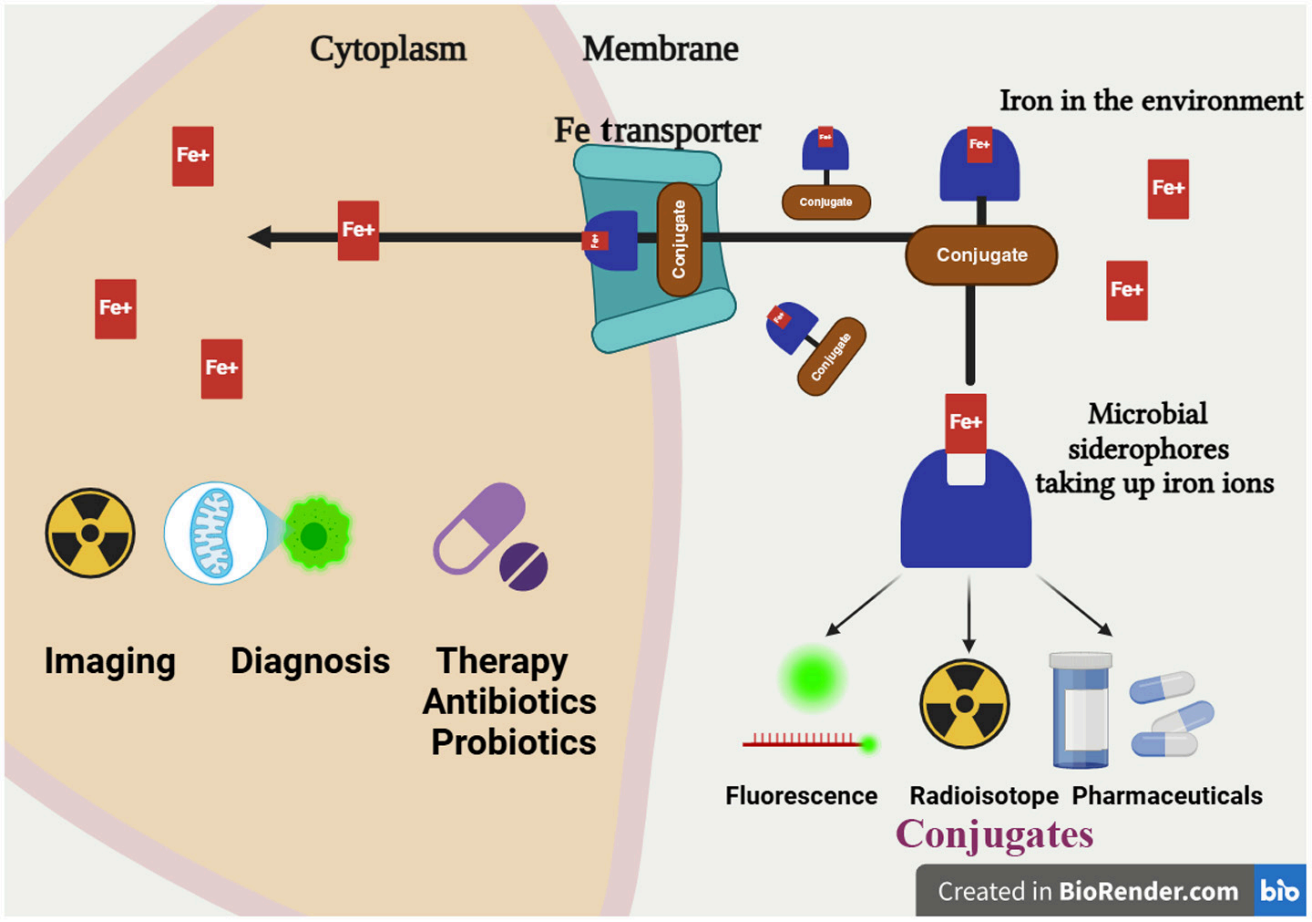
Siderophore conjugates serve as a powerful tool in healthcare applications.

It is imperative to eradicate the infections caused by various pathogens, using either antimicrobials or vaccines. This review has already discussed many aspects of using siderophores as a tool for alternative treatment. The documentation of siderophores as a potential treatment option in medical applications is meagre, particularly in developing vaccines [65,66]. Siderophore-based vaccines are extensively used in making siderophore-based-complex antibiotics to enhance their strength and help treat antibiotic-resistant infectious diseases [67,68]. Hence, a potential vaccine, an antigen cloned with a siderophore-related gene, can help treat or ameliorate several infectious diseases (Fig. 7). It has been noted that the Enterobacteriaceae family is prominently involved in most urinary tract infections (UTIs) in women. The colonization and severity of UTIs are due to specific types of adhesins, siderophores, and toxins produced by uropathogens. Most women are diagnosed with symptomatic UTI and treated with antibiotics like trimethoprim–sulfamethoxazole, cephalosporins, and ciprofloxacin. Gram-negative pathogens have developed a growing antibiotic resistance, resulting in limited antibiotic availability and the need to explore replacement remedies for managing and treating recurrent UTIs. To date, many scientists have developed vaccines based on siderophores to treat these uropathogenic infections, such as yersiniabactin conjugated with bovine serum albumin, which also utilizes aerobactin to check its activity in UTIs. In a study, mice were administered with siderophores conjugated to an immunogenic carrier protein molecule; the siderophore–protein conjugates triggered the adaptive immune response with enhanced activity. Hence, this enabled targeting of bacterial stealth siderophores and protecting against UTIs [69].

A study on ileal Crohn’s disease demonstrated intestinal inflammation due to adherent-invasive *E. coli* (AIEC), leaving limited treatment options to help selectively target AIEC. As iron is an essential micronutrient required by bacterial pathogens, the strategies needed to capture iron have evolved through the environment. The study highlighted immunization to provoke antibodies against these siderophores that are reduced from the AIEC system, interfering with their association with the mucosa and helping lessen colitis in an experimental mice model that was also visualized by flow cytometry to isolate siderophore-specific B cells that are an essential prerequisite for engineering monoclonal antibodies [70].

### Siderophores for molecular imaging of infection

Accurately determining the location and nature of an infection and differentiating it from inflammation is now one of the most challenging tasks in modern medicine. Identifying and diagnosing high-risk patients early and accurately is very important for successful treatment. This highlights the immediate requirement for diagnostic techniques that are both specific and sensitive. Molecular imaging techniques can offer more reliable, nonintrusive, exact, and sensitive detection of infections. This results in enhanced clinical decision-making and a fundamental shift in patient care, leading to improved healthcare results. Radiological imaging techniques, such as computed tomography, magnetic resonance imaging, and ultrasonography, are commonly employed in clinical settings to detect infections. However, it is essential to note that these techniques have significant limitations regarding their ability to identify specific infections accurately.

Optical imaging is a promising method for molecular imaging of infection in the future. However, no optical probes are approved for regular usage in the clinic to detect microbes. In contrast, nuclear imaging techniques such as positron emission tomography and single-photon emission computed tomography have a diverse range of radiolabeled probes (radiopharmaceuticals) with a long history of use in imaging infectious processes in patients. The imaging techniques used in this study involve using radiolabeled leukocytes, anti-granulocyte antibodies, diphosphonates for bone scanning, citrate labeled with gallium-67, and fluorodeoxyglucose labeled with fluorine-18. These probes primarily focus on secondary effects of infection, such as increased blood flow and vascular permeability, activated endothelial manipulation, induced immune response cell, or polymorphonuclear cell migration. However, they have limitations in terms of specificity due to their tendency to affect blood manipulation or induce an immune response [71]. Despite the emergence of breakthroughs, such as radiolabeled antimicrobial peptides, showing promising bacterial infectious treatment, nuclear medicine clinicians are still eagerly anticipating the arrival of superior radiopharmaceuticals that can overcome the current constraints (Fig. 7) [72].

Radiolabeled siderophores seem to be a fascinating collection of molecules that have the potential to meet the criteria for “optimal imaging agent” in molecular imaging with infections. Table 2 provides a concise overview of the various uses of siderophores as imaging agents. They can be created by either introducing a suitable radiometal to the natural siderophore complex through iron exchange or chemically modifying the natural siderophore with a chromophore ideal for optical imaging. In the 1970s and 1980s, researchers conducted initial studies on radiolabeled siderophores, specifically DFO, using gamma-emitting radionuclides such as ^67^Ga and ^111^In. Gallium is an isosteric diamagnetic replacement for Fe(III), resulting in similar affinity constants of numerous siderophores for gallium compared to iron. It was also proven that Ga(III) may quickly replace Fe(III) from siderophores under reduced circumstances. However, without simultaneous reduction of iron, no significant exchange was observed. Emery and Hoffer utilized ^67^Ga to investigate the absorption methods of several siderophores in *U. sphaerogena*. They discovered that this process, which relies on energy, is identical to the uptake mechanism of Fe(III) in the same organism. They hypothesized that the binding of siderophores plays a role in the buildup of ^67^Ga-citrate in inflammatory lesions. Several studies were conducted using ^3^H-, ^14^C-, ^55^Fe-, and ^59^Fe-labeled siderophores to examine the mechanisms of iron transport or siderophore uptake in microorganisms or plants. However, these methods are unsuitable for molecular imaging and cannot detect microbial infections in living organisms [72]. In contrast, the radionuclides ^67^Ga and ^111^In, utilized to investigate nuclear medicine for single-photon emission computed tomography imaging, were conducted by Moerlein and Emery. In the last 10 years, there has been a substantial rise in the use of positron-emitting radiometals in positron emission tomography imaging (Table 2). There has been a significant increase in the use of ^68^Ga due to its capacity to label a wide variety of compounds. This is mainly attributed to the fact that it can be generated via a ^68^Ge/^68^Ga generator system, which has a long shelf-life and is reasonably inexpensive.

**Table 2.** Applications of siderophores as imaging agents in medical diagnosis

Imaging modality	Type of label	Siderophore	Application	Reference
SPECT	^67^Ga	Ferrichrome, ferrichrome A, rhodotorulic acid, triacetylfusarinine C, malonichrome, desferrioxamine	Microbial iron transport	[86]
Fluorescence imaging	Rhodamine B analog, anthracene analog, 7-nitrobenz-2-oxa-1,3-diazole analogs, fluorescein analogs	Ferrichrome, desferrioxamine, pyochelin	Microbial iron uptake and transport; siderophore/iron metabolism	[86,87]
SPECT	^67^Ga, ^111^In	Enterobactin	Ligand for radiopharmaceuticals	[86]
PET	^68^Ga, ^89^Zr	Ferrichrome, ferrichrome A, triacetylfusarinine C, desferrioxamine, desferrioxamine E, coprogen, fusarinine C, ferricrocin	Infection imaging	[88]
SPECT	^67^Ga, ^111^In	Desferrioxamine	Development of novel radiopharmaceuticals; tumor and abscess imaging	[89]

SPECT, single-photon emission computed tomography; PET, positron emission tomography

## Future Challenges in Siderophore Applications

Siderophore biology has emerged as an exciting field in recent years, and new classes and applications have been discovered. It requires further detailed understanding since it is a key component of pathogenicity in infections. Profound expertise and knowledge are essential to identify the mechanism of host–pathogen involving iron molecules. This can provide a proper insight into targeting clinical pathogens and treating them with newer therapeutic options. It can also be used to preserve and process food, improve agricultural products with more iron content, overcome bioaccumulation of iron, and several other bioremediation approaches. The siderophore inhibitory metabolism process must be thoroughly investigated, like biosynthesis, secretion, import, or utilizing their conjugates for specific functions like drug delivery or therapeutic agents to the pathogens. There is a dearth of structural data and correlation with the function and application that needs to be addressed in food, medical, plant growth, and bioremediation research. To draw a solid fundamental application with the conclusion, we must employ different techniques to detect and characterize siderophores by MS, NMR, MS, and ESI. Further study must be emphasized on genetic manipulations for empirical results that benefit their potential applications. The emergence of siderophores in biology as a new area has given novel opportunities for designing innovative methods and delivering drugs to specific sites, more precisely utilizing nanotechnology. Nanoparticles like iron oxide have been used as therapeutic systems to facilitate more target-specific treatment with drug delivery to the appropriate location. A maximal investigation needs to be carried out on the molecular characterization of microbial siderophore metabolism, as each varies with the origin of organisms. The impending future research might help encompass them for fundamental discoveries in various applications involving human health, nutrition, agronomics, and environmental benefits.

## Conclusion

The exploration of microbial siderophore biosynthesis and production has gained the interest of the scientific world due to the diverse applications of siderophores. Increased systematic information and data might help improve disease management in food technology, agriculture, bioremediation, and biosensors by rendering antimicrobial target sites, providing cancer therapy, etc. Siderophore-based unique drugs will likely endure, broadening the clinicians’ and researchers’ arsenal to fight against MDR pathogens. Additionally, nanotechnology can help enable more specific drug delivery systems to the appropriate location; however, more understanding needs to be covered on the aspects of the mechanism, functioning, and specific targets of siderophores involving the same to help the expansion of potential targets.
